# *Bacillus* spores: a review of their properties and inactivation processing technologies

**DOI:** 10.1007/s10068-020-00809-4

**Published:** 2020-10-06

**Authors:** Won-Il Cho, Myong-Soo Chung

**Affiliations:** Department of Food Science and Engineering, Ewha Womans University, Seoul, Republic of Korea

**Keywords:** Spore formation, Germination, Resistance properties, Sporicidal agents, Non-thermal inactivation processing

## Abstract

Many factors determine the resistance properties of a *Bacillus* spore to heat, chemical and physical processing, including thick proteinaceous coats, peptidoglycan cortex and low water content, high levels of dipicolinic acid (DPA), and divalent cations in the spore core. Recently, attention has been focused on non-thermal inactivation methods based on high pressure, ultrasonic, high voltage electric fields and cold plasmas for inactivating *Bacillus* spores associated with deterioration in quality and safety. The important chemical sporicides are glutaraldehyde, chorine-releasing agents, peroxygens, and ethylene oxide. Some food-grade antimicrobial agents exhibit sporostatic and sporicidal activities, such as protamine, polylysine, sodium lactate, essential oils. Surfactants with hydrophilic and hydrophobic properties have been reported to have inactivation activity against spores. The combined treatment of physical and chemical treatment such as heating, UHP (ultra high pressure), PEF (pulsed electric field), UV (ultraviolet), IPL (intense pulsed light) and natural antimicrobial agents can act synergistically and effectively to kill *Bacillus* spores in the food industry.

## Introduction

Many species of spore-forming bacteria are associated with food spoilage (Gould, 2006; Stragier and Losick, 1996). Bacterial endospores, especially *Bacillus* species, are the inactivation target in various forms of food processing. One of the most important microorganisms as significant pathogens in humans or involved in quality damage, *Bacillus* genus such as *B*. *subtilis*, *B. amyloliquefaciens*, *B*. *cereus*, *B*. *licheniformis*, *B*. *pumilus*, and *B*. *thuringiensis*, etc. are a rod-shaped, gram-positive bacterium that are naturally found in soil and vegetation. Specifically, *B. cereus* and *B. anthrax* are representative pathogenic spore forming bacteria, and *B. subtilis*, *B. stearothermophilus* and *B. amyloliquefaciens* are the main bacteria that cause degradation of processed foods. Therefore, killing *Bacillus* spores associated with pathogenicity and deterioration is important for sterilization of processed foods (Higgins and Dworkin, 2012; Leuschner and Lillford, 2003). The representative *Bacillus* spores associated with pathogenicity are *B. cereus*. The pathogenicity of *B. cereus* contamination, whether intestinal or nonintestinal, is intimately associated with the production of tissue-destructive exoenzymes. Among these secreted toxins are four hemolysins, three distinct phospholipases, an emesis-inducing toxin, and proteases (Higgins and Dworkin, 2012; Soni et al. 2016).

*Bacillus* endospores are difficult to control in the food industry because their spores have relatively high resistances to physical and chemical treatments (Ablett et al., 1999; Higgins and Dworkin, 2012; Leuschner and Lillford, 2003; Russell, 1990; Setlow and Setlow, 1995). Dormant bacterial endospores are much more resistant than vegetative cells to common inactivation and disinfection treatments, including heat, radiation, and various chemicals (Amador-Espejo et al., 2014; Anderson et al., 2000; Banksm et al., 1988; Gould, 2006; Peleg and Cole, 2000; Russell, 1990; Setlow and Setlow, 1995).

Processes designed to inactivate *Bacillus* spores in foods need to take this high level of resistance into account. Special attention must also be given to the processing and preserving of foods so that the spores are either inactivated or prevented from undergoing germination and outgrowth. Focusing on the research results of the last 5 years, this review article summarizes related studies on their biochemical and growth properties and the novel inactivation technologies of *Bacillus* spores.

## History of the discovery of spore

### Sporulation

Bacterial endospores were first studied 130 years ago by Cohn and Koch in 1876, independently. Cohn conducted experiments to understand about the new science of bacteriology, and Koch first described the complete sporulation–germination–multiplication–resporulation life cycle of sporeformers in 1888 (Ablett et al., 1999; Atrih and Foster, 2002; Gould, 2006; Leuschner and Lillford, 2003; Stragier and Losick, 1996). Following Koch’s primary observation, detailed description of spore formation using an optical microscope was made by Knaysi (1938) (Ablett et al., 1999; Gould, 2006; Stragier and Losick, 1996). Fitz-James (1960) studied to describe asymmetric septation, engulfment and the sequential formation of the spore with the electron microscopy (Ablett et al., 1999; Leuschner and Lillford, 2003; Setlow and Setlow, 1995). These studies lead to the subdivision of the sporulation sequence into seven distinct stages by Ryter et al. (1966), which became the key basis for sporulation studies and remains so today (Ablett et al., 1999; Gould, 2006). And genetic studies were carried out to investigate for developmental sequence of transformation with numerous sporulation-defective mutants of *B. subtilis* by Errington (1993) and to uncover the controlling sigma cascade by Stragier and Losick (1996) and Sonnenshein (2000) (Ablett et al., 1999; Atrih and Foster, 2002; Gould, 2006; Leuschner and Lillford, 2003; Stragier and Losick, 1996).

### Germination

Powell and Strange (1953) conducted the discovery of high levels of calcium, and determination of the structure of dipicolinic acid (DPA) and the identification of muramic acid in the peptidoglycan fragments that leaked from germinating spores (Ablett et al., 1999; Gould, 2006; Stragier and Losick, 1996). The first genetic studies on germination were initiated by Smith, Moir and their colleagues in the 1980 s, which led to the identification of germination receptors (Ablett et al., 1999; Gould, 2006; Stragier and Losick, 1996). And the trigger’ reactions on spore germination were identified action of germination enzymes, first by Foster and Johnstone (1990) (Gould, 2006; Stragier and Losick, 1996).

Cohn observed that new media supported germination, and Hills (1949) identified l-alanine and some other amino acids (including l-aminobutyric acid, l-cysteine, l-valine and l-leucine) as the specific chemicals that caused germination of the spores of various *Bacillus* species (Ablett et al., 1999; Gould, 2006; Russell, 1990; Stragier and Losick, 1996).

Ribosides (adenosine, inosine, guanosine and xanthosine) were shown to be effective germinants for some types of *Bacillus* spores and to be synergistic with the specific amino acid germinants. Levinson and Hyatt (1966) first discovered the effectiveness of certain sugars and amino sugars (glucose, fructose, 2-deoxyglucose, glucosamine and *N*-acetylglucosamine) as germinants for *Bacillus* spores, and their synergy with amino acids and cations (K^+^) (Atrih and Foster, 2002; Gould, 2006; Russell, 1990).

Mild preheating induced rapid subsequent germination of spores in the presence of specific germinants. Ablett et al. (1999) suggested that the germination mechanism of spore protoplasts by heat activation may be in a glassy state from temperature-induced glass transition (Gould, 2006; Stragier and Losick, 1996).

## Biochemical and growth properties of *Bacillus* spores

### Sporogenesis and germination

Spores are generally formed when the vegetative organism is stressed by limiting the availability of nutrients, and pathogens allow it to survive in a dormant state outside the aerobic or anaerobic environment of the intestine until a new host is colonized (Atrih and Foster, 2002; Higgins and Dworkin, 2012; Russell, 1990).

**Fig. 1:**
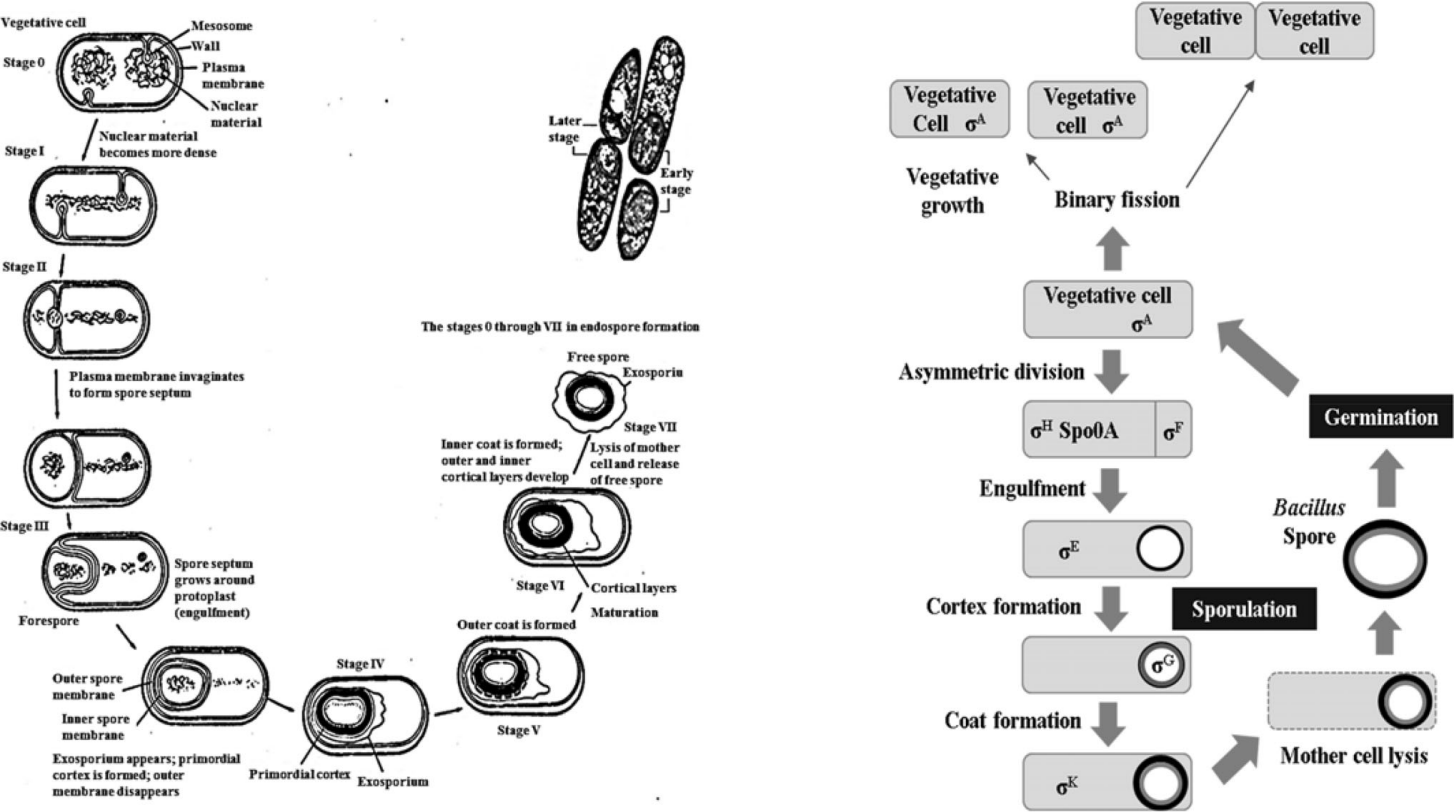
Schematic representation of *B*. *subtilis* spore formation based on morphological mechanism (Atrih and Foster, 2002; de Hoon et al., 2010; Driks, 1999; Higgins and Dworkin, 2012; Gould, 2006; Russell, 1990; Sandra et al., 2014; Sekiguchi et al., 1999; Setlow et al., 2000)

The Gram-positive bacteria *B. subtilis* can initiate the sporulation process under nutrient restriction conditions. The morphological mechanism of the sporulation process (Fig. 1) generally consists of seven stages as follows: cell division is stopped by a spore-formation signal, and the nuclear material is disposed axially into filaments the nuclear material becomes denser in stage I. The plasma membrane then invaginates to form a spore septum in stage II, and completion of DNA segregation occurs concurrently with the invagination of the plasmatic membrane in an asymmetric position, near one pole of the cell, forming a septum. The exosporium appears, the primordial cortex is formed, and the outer membrane disappears sequentially in stages III and IV. Specifically, spores, the septum begins to curve, and the immature spore is surrounded by a double membrane of the mother cell in an engulfment process, similar to phagocytosis, and the smaller fore-spore becomes entirely contained within the mother cell in stages III. The mother cell mediates the development of the fore-spore into the spore in stage IV. Then, the inner and outer proteinaceous layers of the spores are assembled, and the spore cortex composed of a thick layer of peptidoglycan contained between the inner and outer spore membranes is synthesized. Moreover, calcium dipicolinate accumulates in the nucleus in stage IV (Atrih and Foster, 2002; de Hoon et al., 2010; Higgins and Dworkin, 2012; Gould, 2006; Sandra et al., 2014; Sekiguchi et al., 1999; Setlow et al., 2000).

**Fig. 2:**
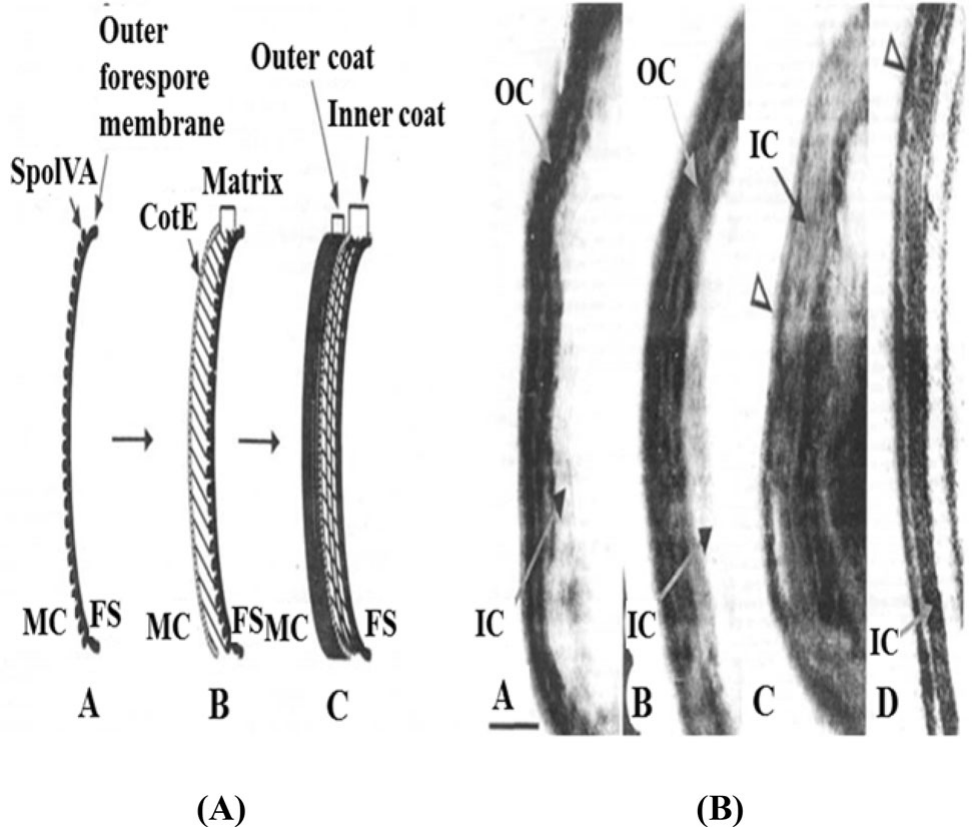
(A-I) Model of the stages of coat formation: [A] SpoIVA localizes to the mother-cell (MC) side of the sporulation septum, of which only an arc is shown; [B] the precoat, consisting of the matrix and the layer of CotE protein, assembles at the forespore (FS) surface, under the direction of SpoIVA; and [C] the inner-coat (IC) proteins assemble into the matrix and the outer-coat (OC) proteins bind the shell of CotE. (A-II) Electron microscopy images of wild-type and mutant spore coats, showing arcs of wild-type [A], AD408 [B], TB50 [C], and TB70 [D] spore coats. The open arrowheads indicate thin remnants of the outer coat. Bar, 100 nm (Meador-Parton and Popham, 2000; Sekiguchi et al., 1999)

The formation of the outer and inner coats continues with the development of outer and inner cortical layers in stages V and VI. The spore coat is synthesized, consisting of proteins deposited by the mother cell, and is arranged in inner and outer layers in stage V. The formation of the coat begins just after the formation of the sporulation septum. At that time the protein called SpoIVA binds at or very close to the adjacent mother cell (Meador-Parton and Popham, 2000). In the second stage, SpoIVA connects the preliminary coat structure, called the precoat, to the mother-cell side of the forespore (Riesenman and Nicholson, 2000). In the last stage, the precoat is a 181-amino-acid protein called CotE with a stretch of acidic residues between amino acids 150 and 169 (Heo and Cho, 2002). After the precoat is assembled, the inner coat protein penetrates into the backbone formed by the precoat and the outer-coat protein is assembled around the shell of the CotE protein (Fig. 2) (Meador-Parton and Popham, 2000; Sekiguchi et al., 1999). Spore maturation occurs during this stage, and the spores become resistant to heat and organic solvents in Stage V. Finally, lysis of the mother cell and release of a free mature spore occurs in stage VII (Fig. 1) (Atrih and Foster, 2002; de Hoon et al., 2010; Higgins and Dworkin, 2012; Gould, 2006; Russell, 1990; Sandra et al., 2014).

**Fig. 3:**
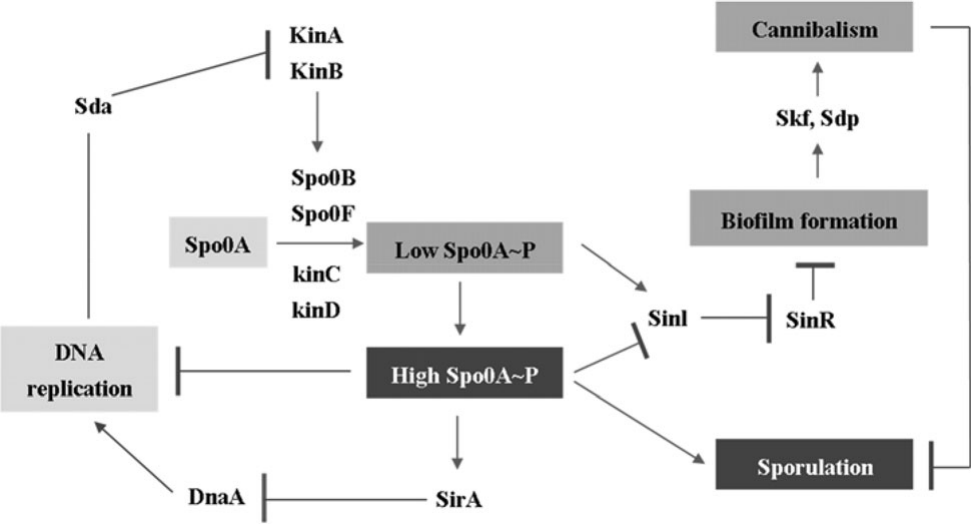
Schematic representation of *B*. *subtilis* spore formation based on biochemical mechanism (Atrih and Foster, 2002; Driks, 1999; Higgins and Dworkin, 2012; Irene and Kumaran, 2014; Gould, 2006; Russell, 1990; Sekiguchi et al., 1999; Setlow et al., 2000)

Comparative studies have described the biochemical mechanisms of spore formation based on cascade theory (Driks, 1999; Setlow et al., 2000). In this mechanism, the signal for spore formation is first transferred by the phosphoryl system with a multicomponent regression function. The continuous expression of a spore-forming gene called a sigma (σ) cascade controls the mechanism of spore formation. The expression of the gene related the spore formation is controlled by six kinds of σ factor (designated as A, H, F, E, G, and K): the A factor is related to housekeeping and early sporulation on vegetative-cell, the H factor induces the expression of gene-related spore formation by sporulation regulatory genes (Spo0A–P), which is the beginning stage. And the E factor is involved in the early mother cell gene expression, the F factor is concerned with early forespore gene expression, the K factor is related to the formation of the spore-coat proteins, and the G factor induces the gene with ultraviolet (UV)-radiation resistance (Fig. 3) (Driks, 1999; Irene and Kumaran, 2014; Sekiguchi et al., 1999; Setlow et al., 2000).

Recently, Higgins and Dworkin (2012) reported in detail the morphological changes during the sporulation process based on the activation of different sigma specific transcription factors (H, F, E, G and _K) in each spore compartment. First, high levels of H and Spo0A trigger sporulation and lead to the expression of pro-F, and then F is activated only in the forespore by asymmetric division. The SpoIIR protein is produced under the control of F and secreted by the forespore, and activates the proteolytic processing of pro-E → E in the mother cell. After engulfment is complete, a signal produced in the mother cell under the control of E enters the forespore via the SpoIIIAH/SpoIIQ pore and activates G. Finally, the SpoIVB protein produced under the control of G in the forespore activates pro-K → K proteolytic processing in the mother cell via an interaction with the SpoIVFA/SpoIVFB/BofA complex (Fig. 3). (Atrih and Foster, 2002; Higgins and Dworkin, 2012; Irene and Kumaran, 2014; Gould, 2006; Russell, 1990).

A summary of the core mechanisms of spore formation is as follows. The biochemical mechanism underlying the formation of *Bacillus* spores has been explained using sigma (σ) cascade theory. The continuous expression of spore-forming gene is controlled by six kinds of σ factor (designated as A, H, F, E, G, and K).

Spores can remain dormant for long periods of time and retain significant resistance to environmental damage such as heat, radiation, toxic chemicals, and extreme pH. Spores of *Bacillus* species can remain in their dormant and resistant states for years, but when exposed to specific nutrients, spores can return to life within minutes of germination and out-growth. Germination is generally triggered by the presence of nutrients, including amino acids, sugar, and nucleosides (Paidhungat et al., 2001). The process consists of several events in which the exact temporal order has not been clearly determined.

**Fig. 4:**
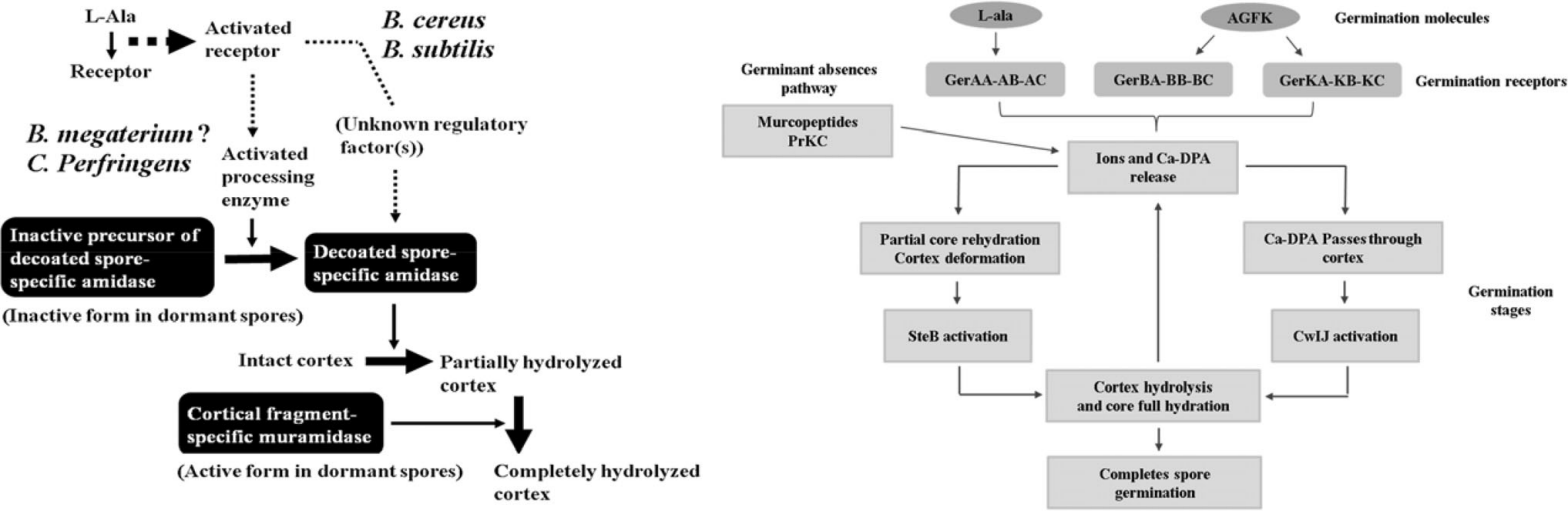
Model of *Bacillus* spore germination. Nutrient-induced spore germination has been divided into stage I, which consists of events that occur in the absence of cortex hydrolysis, and stage II, which consists of all subsequent events, including cortex hydrolysis (Gould, 2006; Moriyama, 1998; Paidhungat et al., 2001; Paredes-Sabja et al., 2011; Sandra et al., 2014; Sekiguchi et al., 1999; Setlow, 2014; Zhang et al., 2012; 2013)

Spore germination induced by nutrients has been divided into stage I, which consists of events that occur in the absence of cortex hydrolysis, and stage II, which consists of all subsequent events, including cortex hydrolysis (Fig. 4) (Gould, 2006; Moriyama, 1998; Paidhungat et al., 2001; Paredes-Sabja et al., 2011; Russell, 1990; Sandra et al., 2014; Setlow, 2014). Some events related to germination at stage I, called activation, occur in the spore core and rehydrate the dehydrated cytoplasm of the spores and excrete large amounts of stored DPA (pyridine-2,6-dicarboxylic acid or dipicolinic acid) and contains divalent cations, Ca^2^^+^ is dominant. It is currently thought that the interaction of nutrients and their receptors in the inner membrane of a spore changes the permeability of that membrane. The most common, initial step in the germination process is the recognition of small molecule germinants by germination (Ger) receptors. Ger receptors are inner-membrane heterogeneous complexes formed by three distinct protein products such as the A-, B-, and C-subunits (Ross and Abel-Santos, 2010). Specifically, the initiation or triggering process in response to nutritional replenishment that occurs when the germinating molecules such as low-molecular-weight amino acids, sugars, and purine nucleosides, are sensed by germination receptors (GRs) located in the inner membrane of the *Bacillus* spore. These receptors include, gerA, gerB, or gerK, and the germinating molecules bind these receptors. l-alanine acts through the gerA receptor, and a mixture of l-asparagine, fructose, glucose and KCl (AGFK) bind the gerB and gerK receptors (Fig. 4). (Gould, 2006; Paredes-Sabja et al., 2011; Sandra et al., 2014; Setlow, 2014; Zhang et al., 2013; Zhang et al., 2012). And this phenomenon leads to the release of DPA and cations from the spore core with water absorption, and then leads to spore germination in the stage II (Fig. 4) (Gould, 2006; Moriyama, 1998; Paidhungat et al., 2001; Paredes-Sabja et al., 2011; Sandra et al., 2014; Sekiguchi et al., 1999; Setlow, 2014). Specifically, DPA (pyridine-2,6-dicarboxylic acid) is degraded and released, the rehydration of the spore core in the stage II is followed. Rehydration allows the initiation of protein mobility and reactivation of bio-chemical processes during outgrowth (Zhang et al. 2010).

The hydrolysis reaction in the spore cortex associated with stage II is not necessary for germination events in the spore core. The cortex hydrolysis is absolutely necessary for the subsequent steps in germination that, including the initiation of spore metabolism and the growth of the germinated spore. The energy needs in the early stage of growth are met by the catabolism of compounds such as 3-PGA (3-phosphoglycolic acid) stored in the core, and all events that make up the germination process occur without energy metabolism PGM. Cortex hydrolysis is another stage of germination that has been studied in some detail. Hydrolysis of the cortex during spore germination involves several spore enzymes called cortex-lytic enzymes (CLE). Two CLEs, named SleB and CwlJ, have been implicated in cortex hydrolysis in *B. subtilis* during nutrient-induced spore germination (Paidhungat et al., 2001).

Spore germination is most commonly triggered by specific nutrients, but it can also be induced by certain chemicals that are not nutrients and by physical treatment such as ultra-high pressure, ultrasound, and electric fields (Moriyama, 1998; Paidhungat et al., 2001; Russell, 1990; Sekiguchi et al., 1999; Setlow, 2014). The best-known example of a nonnutrient chemical germinant is a 1:1 chelate of Ca^2+^ and DPA, which triggers the germination of spores in many species of endospore-forming bacteria. The nutrient germinants bind to specific spore receptors that are encoded by the three expressed members of the *gerA*family of operons. As a result, Ca^2+^ and DPA are expected to trigger cortex hydrolysis, but it is not known whether the same two CLEs are used in the process (Driks, 2002; Moriyama, 1998; Paidhungat et al., 2001; Russell, 1990; Setlow, 2014). It is very interesting that the mechanisms of nutrients and Ca^2+^ are different and that DPA causes spore germination at least at the receptor-mediated stage (Gould, 2006; Moriyama, 1998; Paidhungat et al., 2001; Russell, 1990; Setlow, 2014).

Spore coat hydrolysis allows the emergence of the incipient vegetative cell. Outgrowth is the transition of the germinated spore to a growing cell, during which time cell division occurs. During the first stage of outgrowth, ATP is generated through the conversion of 3-phosphoglycerate stored in the spore core. At a later stage, the outgrowing spores are converted to the use of extracellular nutrients (Zhang et al., 2010). Initiation of etching and outgrowth of the coat layer typically occurred within 1–2 h and 3–7 h, respectively. During germinant absences, the germination *of Bacillus* spores may also be triggered by extremely low concentrations of muropeptides. Muropeptides are produced by the degradation of peptidoglycans that comprise the cell wall of the majority of bacteria and spore coats (Zhang et al., 2013; 2012).

A summary of the core mechanisms of germination is as follows. Germination occurs in the spore core and includes rehydration of the spore’s somewhat dehydrated cytoplasm and excretion of a large amount of stored DPA (pyridine-2,6-dicarboxylic acid) and divalent cations, predominantly Ca^2+^. Cortex-lytic enzymes have been implicated in hydrolysis of the cortex during spore germination.

### Susceptibility and resistance

There are many factors that determine the resistance properties of spores, including the thick proteinaceous coat, low water content and high levels of DPA and divalent cations in the spore core (Table 1) (Atrih and Foster, 2002; Driks, 2002; Riesenman and Nicholson, 2000).

**Table 1:** Comparison of the structures of a bacterial spore, vegetative cell, and virus

Structure	Vegetable cell (*B. subtilis*)	Bacterial pore (*B. subtilis*)	Virus (influenza)	Reference
Photograph				Russell (1990)
Schematic				Atrih and Foster (2002)
Description	Cell wall: Peptidoglycan, glucan, mannan Rigidity and shape (0.1–0.4 μm) Cell membrane: Phospholipid bilayer, protein Cytoplasmic membrane Permeability barrier	Exosporium: Glucose, lipid, protein Coats: Protein (80%), chemical Resistance (agent, ozone) Double membrane: Phospholipid Cortex: Peptidoglycan, Ca^2+^, DPA Heat and UV resistance Core: DNA with SASP, Ca^2+^	Envelope: Glucose, protein, lipid Capsid: Protein coat Capsomers (polypeptides) Core: RNA, virus genome	Driks (2002) Riesenman and Nicholson (2000)

The extreme outer layer of *Bacillus* spores, exosporium, consists of glucose, lipids and proteins (Atrih and Foster, 2002; Driks, 2002; Russell, 1990). The exosporium constitutes the first barrier between the spore and the environment. It may contribute to protection against damaging macromolecules such as hydrolytic enzymes and antibodies. And also, the exosporium provides resistance to oxidative stress generated by macrophages (Atrih and Foster, 2002; Driks, 2002; Riesenman and Nicholson, 2000).

The double membrane of *Bacillus* spores surrounding the spore cytoplasm contains proteins and lipids essential for membrane structure. Unlike the outer membrane, the spore’s inner membrane is a strong permeable barrier to many chemicals that damage DNA (Atrih and Foster, 2002; Driks, 2002; Russell, 1990).

The spore coating provides the first line of defense against chemical treatment and consists of two layers of proteins that are resistant to mechanical shocks such as high pressure and electrical energy (Amador-Espejo et al., 2014; Cho et al., 1999; Hayakawa et al., 1998; Marquez et al., 1997; Oh and Moon, 2003). The spore coat of *B. subtilis* is a triple-layered structure consisting of an outer coat, an inner coat (crust), and an electron-diffuse undercoat as revealed by electron microscopy (Soni et al., 2016). The spore coat is composed of proteins and glycoproteins and acts as a filter for many molecules such as enzymes, nutrients, and it detoxifies chemicals related to cause damage for spore (Driks, 2002; Setlow, 2006).

The layer under the spore coat consists of a thick layer of peptidoglycan, called the cortex, which covers the core and protects it from chemical damage by organic solvents (Riesenman and Nicholson, 2000). The structure of the *Bacillus* spore contains the peptidoglycan cortex that protects the spore’s inner membrane, which is an important barrier stopping the entry of small hydrophilic molecules into the central spore core (Atrih and Foster, 2002; Jeffrey et al., 2003). The cortex is composed of a matrix structure constructed from peptidoglycan, glycopeptide (spore mucopeptide), and Ca^2+^, and it is resistant to heat and UV radiation (Sun et al., 2016). The cortex functions to accept the germinant and induce the disassembly of the cortex by a hydrolysis enzyme in the middle of spore cores and coats (Moriyama, 1998).

The layer beneath the germ cell wall is an inner membrane that acts as a barrier to chemicals, protecting the central core (Setlow, 2006). Strong resistance to heat and chemical treatment of *Bacillus* spores is due to the unique structure and properties of the core (Atrih and Foster, 2002; Driks, 2002). The central core consists of ribosomes, inert enzymes, and small acid-soluble binding proteins (SASP) that protect the DNA, which is present in the form of a chromatin network (Setlow, 2014**).** And also, this core is similar to the nucleus of vegetable cells from the functional aspect, but the DNA of core contains SASP, DPA, Ca, Mg, and Mn at high concentrations to produce a high resistance to heat denaturation and radiation. The dry state of the core caused by the contractile cortex mechanism is another important factor in increasing the heat resistance of *Bacillus* spores (Riesenman and Nicholson, 2000; Russell, 1990).

As a recent related study, the heat resistance of *B. subtilis* spores was investigated at the genetic level. Deletion of the mobile genetic elements known as spoVA2mob reduces heat resistance in *B. licheniformis*, *B. subtilis* and *B. amyloliquefaciens* strains, whereas insertion has been reported to improve heat resistance (Soni et al., 2016).

A summary of the resistance properties of a spore is as follows. Many factors determine the resistance properties of a spore against heat, chemical and physical treatment, including its thick proteinaceous coat, low water content, and high levels of DPA and divalent cations in the spore core.

## Physical inactivation methods for *Bacillus* spores

### Conventional heat processing

Appert (1810) demonstrated that the heating food in sealed container could keep shelf stable for long periods at room temperature, and Bigelow et al. (1920) made the first mathematical model related to thermal processing as log-linear spore inactivation kinetics (Gould, 2006; Leuschner and Lillford, 2003).

*Bacillus* species with endospores are present in various spicy vegetables such as garlic, ginger, and onion. High-temperature treatment in a retort can be used to sterilize heat-resistant spores. *Bacillus* species are often present in raw milk and play an important role in the spoilage of milk and milk-based products, and the use of ultra-high-temperature (UHT) processing can inactivate intact spores so as to achieve a long shelf life at room temperature (Atrih and Foster, 2002; Crielly et al., 1994; Gould, 2006).

Heat-resistant microorganisms such as *B. subtilis* spores derived from the soil may be present in soy milk, and conventional long-term high-temperature treatment of soy milk provides sufficient inactivation for these spores. However, heating processes involving retort and UHT negatively affect taste, color and texture quality due to protein denaturation and decomposition of nutrients at high temperatures of 121–135 °C (Crielly et al., 1994; Leuschner and Lillford, 2003).

The mechanism of resistance of spores to wet heat is unknown, but can be multicomponent, such as spore protoplasts with low levels of water. In addition, mineralization of spores contributes to heat resistance as does calcium DPA. Contrary to a lack of understanding of the mechanism of wet heat resistance, small DNA-binding acid-soluble proteins are a major cause of dry heat resistance (Atrih and Foster, 2002; Crielly et al., 1994; Leuschner and Lillford, 2003).

### Novel heat processing

The ohmic heating based on electric resistance heating was reportedly used to inactivate micro-organisms in milk in 1919 (Anderson and Finkelstein, 1919). Today, various studies using low frequency alternating current of 500 Hz–20 kHz instead of commercial alternating current of 50–60 Hz were carried out and conditions that can be applied to some products such as paste sauce and fish cake have been established. Ohmic heating with low frequency alternating current is useful because of the increased stability and energy efficiency (Uemura et al., 2010).

Until recently, ohmic heating was generally thought to kill microorganisms through the thermal effect of uniform and rapid heat generation. However, more and more evidence suggests that non-thermal effects may occur (Somavat et al., 2012). Ohmic treatments at frequencies of 60 Hz and 10 kHz were compared with conventional heating at 121, 125 and 130 °C for four different holding times. Both ohmic treatments showed a general trend of accelerated *Geobacillus stearothermophilus* spore inactivation. It is assumed that under high temperature conditions, electric field oscillations of polar dipicolinic acid molecules (DPA) and spore proteins can lead to accelerated inactivation. However, there is very little literature on the specific non-thermal effect of electricity on bacterial spores during ohmic heating. Efficient biological verification of ohmic heating to realize the full potential of the process requires new analytical and interpretation methods. If there is a non-thermal effect of electricity on ohmic heating, it can potentially improve quality by reducing process severity (Somavat et al., 2012).

Uemura et al. (2010) studied the effect of inactivation of *B. subtilis* spores in soymilk with radio-frequency heating (Uemura et al., 2010). When processing soymilk at a frequency of 28 MHz, the following results were obtained. Radio-frequency heating processing reduced *B. subtilis* spores in soybean milk by four-logarithmic orders at an outlet temperature of 115 °C. When processing soymilk, the Teflon film covering the electrode effectively prevented scaling. Tofu made with radio-frequency heated soymilk had a higher breaking strength than tofu made with conventional heated soy milk. It was concluded that radio-frequency heating could be used to improve soybean safety and tofu quality (Uemura et al., 2010).

### Non-thermal inactivation processing

Recent attention has been focused on nonthermal microbial inactivation methods that are based on various types of physical energy such as high pressures, ultrasound, high-voltage electric fields, and cold plasma for inactivating food-borne enteropathogenic bacteria and spoilage fungi related to the deterioration of quality and safety (Cho et al., 1999; Hayakawa et al., 1998; Marquez et al., 1997; Mendes-Oliveira et al., 2019).

However, endospore-forming bacteria such as *B. subtilis* exhibit resistance to physical treatments. Many new methods have been studied recently for the purpose of overcoming the resistance of some spores to the nonthermal inactivation methods (Table 2) (Cho et al., 1999; Hayakawa et al., 1998; Marquez et al., 1997; Moreau et al., 2000; Sawai et al., 1997; Vaid and Bishop, 1998; Warrimer et al., 2000). One study investigated the killing of bacterial spores using a high-voltage pulsed electric field (HVPEF) (Marquez et al., 1997). The results indicated that *Bacillus* spores were damaged when the electric field strength was ≥ 35 kV/cm. The basic mechanism of inactivation of spores was explained by pulse polarity, and ions in the spore cortex would become a shielding layer that prevented radiation from penetrating the core, and the cortex was destroyed by ionic interfacial polarization; however, the exact mechanism was not clear (Marquez et al., 1997). In another study, a method associated with concentrated high-intensity electric field (CHIEF) developed by the University of Minnesota has considerable potential as a commercial process for non-thermal pasteurization of fresh liquid foods (Deng et al., 2013). The average (± standard deviation) of microbial inactivation after subjecting milk samples to a single pass through CHIEF was 2.95 (± 0.35), 2.75 (± 0.25), and 0.18 (± 0.15) log CFU/ml for *Salmonella*, *L. monocytogenes* and spore of *B. cereus*, respectively. The inactivation effect of single pass treatment for *Bacillus* spores was less, but additional passes are expected to result in more bacterial reduction (Deng et al., 2013).

**Table 2:** Summary of novel physical methods for the inactivation of *B*. *subtilis* spores

Method		Mechanism	Effect on *B. subtilis* spores	Equipment	References
Indirect	1. Far-infrared irradiation	Rapid heat activation of spores	Increased germination	Irradiation system	Sawai et al. (1997)
	2. Ohmic heating	Tyndallization (spore germination) increased by applying an electric current	Increased germination	Ohmic heater	Cho et al. (1999)
	3. Microwave irradiation	Explosion of internal pressure generated within the core		MDS-2100 microwave generator	Vaid and Bishop (1998)
Indirect + direct	4. High-voltage pulsed electric field	1. Cortex destruction by pulse polarity 2. Rapid germination of spores	10^1^–10^3^ inactivation	HVPEF system (50 kV/cm)	Marquez et al. (1997)
Direct	5. UV excimer laser irradiation	Core DNA disruption	10^4^–10^6^ inactivation	KrF excimer laser	Warrimer et al. (2000)
	6. Manothermosonication	1. Cavitation mechanical stress 2. H- and OH- free radicals	10^1^–10^3^ inactivation	Resistometer	Sagong et al. (2013)
	7. High-frequency ultrasound	Imperceptible damage to the spore coat (e.g., “pinholes”)	10^2^ inactivation (*B. globigii*)	Ultrasound disruptor	Sagong et al. (2013)
	8. Flowing afterglow of plasma	Break bonds in the coat materials and damage the DNA helices, slow combustion of the spore material	10^6^ inactivation (50 °C for 40 min)	Plasma sterilizer with MW afterglow	Moreau et al. (2000)
	9. Rapid decompression from a high pressure	Physical breakdown of spore coat increases the permeability to water	10^3^–10^4^ inactivation	Link-Motion System, E.G. seal MT	Hayakawa et al. (1998)

Hayakawa et al. (1998) reported the use of rapid decompression to inactivate heat-tolerant spores of *B*. *stearothermophilus* IFO 12550. Below 400 MPa, heat-resistant spores such as *B. stearothermophilus* could not be sterilized using a simple pressing method (Amador-Espejo et al., 2014; Hayakawa et al., 1998). However, when 200 MPa pressure was applied at 75 °C for 60 min, the spore killing rate was 4 log CFU/ml. This inactivation process increased permeability to water at high pressure and high temperature due to the physical destruction of the spore coat (Amador-Espejo et al., 2014; Hayakawa et al., 1998).

UV irradiation is effective at killing spore forming bacteria that contaminate the surface of various substances. It has been established that the inactivation of cells by UV irradiation is mainly due to the fatal effect on DNA. Exposure to UV radiation can cause a number of detrimental effects such as abnormal ion flow, increased cell membrane permeability and depolarization of the cell membrane (Cho et al., 2012; Gayán et al. 2013; Sun et al., 2016). UV_254_ showed the strongest inactivation effect, the level of *B. subtilis* spores decreased by about 3.6-log cycle after 3 min of exposure, and 2.5-log reduction in spores after 3 min when exposed to UV_185_ (Cho et al., 2012).

The intense pulsed light (IPL) is one of the non-thermal treatment technologies that applies intense, short-term light pulses to the food surface. The spectrum used by IPL is similar to sunlight (170–2600 nm), but the intensity of light is 20,000 times stronger. This light can be applied to food surfaces or packaging materials to effectively kill microorganisms with a 3–6 log reduction in *Bacillus* spores. Although the mechanism of inactivation of IPL has not been fully elucidated, it is generally accepted that the main lethal action of IPL can be attributed to photothermal and/or photochemical mechanisms associated with chemical modification and DNA cleavage (Anderson et al., 2000; Cho et al., 2012; Setlow, 2006).

## Chemical inactivation methods for *Bacillus* spores

### Chemical sporicidal agents

Thermal processing of food is a widespread, effective and relatively inexpensive method to prevent for damaging food by decaying microorganisms and undesirable enzymatic reactions. However, heat treatment can reduce nutrient content, modify organoleptic qualities such as fresh, and limit the type of package material available to withstand high processing temperatures. For this reason, alternative processes are being developed using chemicals to minimal changes to the fresh properties of food (Brantne et al., 2014; Kim et al., 1999; Sagripanti and Bonifacino, 1996).

The basic mechanism of the sporicidal action of chemical agents is not well known, mainly due to the complex nature of bacterial spores. The spore surface is hydrophobic, and the complete spore presents several sites at which interaction with chemical agents is possible, such as the inner and outer spore coats, the cortex, spore membranes, and the core (Brantne et al., 2014; Kim et al., 1999; Stragier and Losick, 1996). In addition, the complex structure and composition of spores plays an important role in chemical resistance. The spore has a structure very different from a cell in growth with a number of features and components of the original spores (Kim et al., 1999; Russell, 1990; Sagripanti and Bonifacino, 1996).

Comparatively few antimicrobial agents are actively sporicidal. Even quite powerful bactericides may inhibit spore germination, outgrowth, or both processes; that is, be sporostatic rather than sporicidal (Table 3) (Banksm et al., 1988; Chaibi et al., 1996; Heo and Cho, 2002; Kida et al., 2003; Ko and Kim, 2004; Russell, 1990; Sagripanti and Bonifacino, 1996). The most important chemical sporicides are glutaraldehyde, formaldehyde (both liquid and vapor forms), chorine-releasing agents, peroxygens, ethylene oxide, and ozone (Table 3) (Banksm et al., 1988; Chaibi et al., 1996; Heo and Cho, 2002; Kida et al., 2003; Ko and Kim, 2004; Russell, 1990; Sagripanti and Bonifacino, 1996). Sporicidal treatment always takes considerably longer than treatment for vegetative cells and requires higher concentrations when chemicals are involved.

**Table 3:** Agents exhibiting bactericidal and sporicidal activities

Antibacterial agents	Sporicidal concentration (%wt/vol)	Structures targeted	Comment	References
Chlorocresol	> 0.4	Cortex		Russell (1990)
Cresol	> 0.5			Russell (1990)
Phenol	> 5.0			Russell (1990)
Phenylmercuric nitrate	> 0.02			Sagripanti and Bonifacino (1996)
Chlorhexidine diacetate	> 0.05^a^	Cortex	UDS^b^ spores more sensitive than “normal” spores	Sagripanti and Bonifacino (1996)
Cetylpyridinium chloride	> 0.05^a^	Cortex		Russell (1990)
Glutaraldehyde	2.0	Cortex	UDS^b^ spores highly sensitive	Sagripanti and Bonifacino (1996)
Formaldehyde	4–8			Russell (1990)
Hydrogen peroxide		Core	Varies with species	Russell (1990)
Hypochlorite	20 ppm	Cortex	USD^b^ spores highly sensitive	Sagripanti and Bonifacino (1996)
Polylysine	0.5	Coat, Cortex		Heo and Cho (2002)
Lysozyme		Cortex	UDS^b^ spores highly sensitive	Kida et al. (2003)
Sodium lactate				Banksm et al. (1988)
Poly fatty acid ester				Chaibi et al. (1996)
Protamine		Core		Kida et al. (2003)
Thiamine dilaurylsulfate	1.0	Coat, Cortex		Kida et al. (2003)
Nisin		Core		Kida et al. (2003)
Ethanol		Core		Ko and Kim (2004)

*It means that exhibiting bactericidal and sporicidal activities of the chemical agents can be affected by pH, temperature and treatment time in sterilization of *Bacillus* spores

^a^Not sporicidal at this concentration at ambient temperatures

^b^UDS (Urea + Dithiothreitol + Sodium lauryl sulfate at alkaline pH) : the treatment of UDS were used for achieving such spores with solubilizing of coat proteins

Some food-grade antimicrobial agents such as protamine, polylysine, sodium lactate, poly fatty acid ester, essential oils and organic acids exhibit sporostatic and sporicidal activities (Chaibi et al., 1996; Heo and Cho, 2002; Kim et al., 1999; Sagripanti and Bonifacino, 1996). Protamine extracted from the spermary of fish inhibits the functions of peptidoglycan, DNA, RNA, protein synthesis, and ATP related to spore growth (Kida et al., 2003; Kim et al., 1999). Inactivation of spores by protamine improves when combined with heat treatment (Kida et al., 2003; Kim et al., 1999). Polylysine exerts an inhibitory effect on spore germination through its surfactant function (Heo and Cho, 2002; Ko and Kim, 2004). And sodium lactate, glycine, lysolecithin, poly fatty acid ester, l-phenylalanine, essential oils, and l-serine can inhibit the growth of spores due to binding between the polar groups in the spore coat and the hydrophobic groups of surfactant components (Oscroft et al., 1990; Ruth, 2001; Setlow et al., 2002a; 2002b). Organic acids such as lactic acid are best suited for the development of novel antibacterial substances for inactivating *B. subtilis* spores. The killing effect of organic acids such as lactic acid, citric acid and acetic acid on intact spores at low pH conditions was considered to be due to unstable growth environments by changes in ionic composition (Oscroft et al., 1990; Ruth, 2001; Setlow et al., 2002a; 2002b).

### Sporicidal agents of plant original

Some chemical food preservatives have a long history of safe use, but there are reports that sensitive individuals sometimes develop allergic reactions and can produce carcinogenic byproducts. This has raised concerns about the harmful effects that preservatives can have on health. This has led to a revival of interest in antibacterial compounds of plant origin.

More than 1300 plants are known as potential sources of antibacterial agents. Antibacterial plants that could be used as preservatives can be divided into several different categories, including phenolics, polyphenol, guinones, flavones, flavonoids, flavonols, tannins, coumarins, terpenoids, alkaloids, lectins, and polypeptides. Many plant-derived antimicrobial compounds exhibit a wide spectrum of activity against bacteria, which has led to suggestions that they could be used as natural preservatives in foods (Kim et al., 1999; Kim and Shin, 1997; Nishina et al., 1995). The tannin, polyphenol, theaflavin and catechin in tea extract, and caffeic acid were effective inhibitors of the germination of *Bacillus* spores (Kim and Shin, 1997; Nishina et al., 1995). Germination inhibition was presumed to result from the effects of eugenol, thymol, citral, cinnamaldehyde, camphor, limonene, carvone, cymene, and capsaicin, but the underlying inhibitory mechanism was not adequately described (An et al., 1999; Chaibi et al., 1996; 1997; Choi et al., 1997).

Many surfactants with hydrophilic and hydrophobic properties can inactivate various types of *Bacillus* spores. The crude extracts of *Torilis japonica*, *Gardenia jasminoides*, *Plantago asiatica*, *Fritillaria* species, and *Arctium lappa* with surfactant components showed particularly high sporicidal activities, reducing spore counts by about 2 log CFU/ml (Cho et al., 2015; Kim et al., 1999; Setlow et al., 2000). In addition, the decreasing of 1–2 log CFU/ml on the number of spores was achieved by treating each surfactant component such as 1% (w/w) concentration bornyl acetate, geranyl acetate, pinene, p-cymene, camphene, citral, 2,3-dihydrobenzofuran, polylysine and thiamine dilaurylsulfate (Table 4) (An et al., 1999; Chaibi et al., 1997; Cho et al., 2015; Choi et al., 1997). It has also been found that hydrophobic surfactants were more effective at killing *B. subtilis* spores than hydrophilic surfactants. Furthermore, the sporicidal effects of surfactants such as geranyl acetate and c-terpinene were found to be significantly enhanced in the presence of a germinant, because l-alanine and synergistic cofactors (e.g., K ions) trigger cortex hydrolysis in spores (Cho et al., 2015; Gould, 2006; Russell, 1990).

**Table 4:**
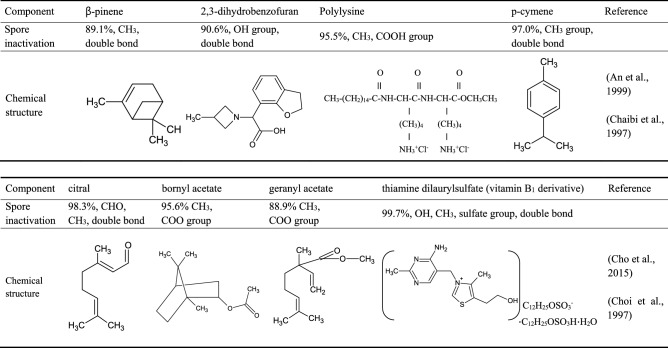
Various surfactant components exhibiting sporicidal activity against *B. subtilis* spores

In addition, the method of chemical inactivation using synthetic preservatives is expected to decrease gradually, taking into account the tendency of consumers’ health preferences. Therefore, there is a need for research on a method for sterilizing *Bacillus* spores related to pathogenicity and quality damage using natural anmicrobial agents. In addition, various studies on effective environmentally friendly physical methods using electricity, pressure, and light energy that can replace chemical inactivation are important.

## Inactivation by combination of physical and chemical methods

### Inactivation using combination of physical energy

Consumers’ demands for safe foods with good taste and high nutritional value are increasing. Innovative thermal and non-thermal methods associated with microbial inactivation are alternatives to preparing safe foods that are fresh and minimally processed. Combining physical methods based on various types of energy is more effective than applying a single energy type for inactivation of *Bacillus* spores.

The efficacy of ultrasound treatment is often increased by combining it with other physical or chemical lethal factors that help to shorten the treatment time and improve quality retention. The inactivation of *B. subtilis* spores by ultrasound waves under pressure (manosonication [MS]) and by combined mild heating and MS treatment (manothermosonication) has been studied (Raso et al., 1998; Sagong et al., 2013). Combining heating at 70–90 °C with MS treatment at 20 kHz, 300 kPa, and 117 μm for 6 min had a synergistic effect on spore inactivation as 90–99.9% disruption for *B. subtilis* spores. The results suggested that the mechanical effects of ultrasound were probably responsible for the damage to *Bacillus* spores and the associated loss of viability (Raso et al., 1998; Sagong et al., 2013).

In addition, combined pressure and ohmic heating sterilization is a new technology developed by Ohio State University to preserve food while maintaining the quality properties desired by consumers (Park et al., 2013). The pressure-ohmic-thermal sterilization (POTS) for *Bacillus* spores was treated at 50 V/cm, 600 MPa and 105 °C using a laboratory-scale high-pressure processor. *B. amyloliquefaciens* spores suspended in 0.1% NaCl solution (pH 7.0) were inactivated by 4.6 log for a retention time of 30 min (Park et al., 2013).

The result of invesgating for a case study on a inactivation method for Bacillus spore by combines of pulsed electric field (PEF) and heating, a 5-log inactivation of *B. subtilis* spores in milk has been reported to attain by preheating the milk to 95 °C, followed by an increase of temperature to 120 °C by PEF (130 kJ/kg) treatment (Soni et al. 2016). And a 3-log reduction of *B. cereus* spores was achieved with a field strength of 35 kV/cm combined with heat treatment (50 °C) in skim milk and a 6-log reduction was observed at 65–75 °C.

Recently, studies on the inactivation of Bacillus spores using superheated steam have been conducted. Specifically, superheated steam (SHS) was used alone or combined with UV-C irradiation for inactivation of *B. cereus* spores inoculated on stainless steel coupons. In order to effectively inactivate *B. cereus* spores only with SHS treatment, a temperature higher than 250 °C was required, but a synergistic bactericidal effect occurred due to sequential treatment of SHS before or after UV-C irradiation (Kim et al., 2020).

Microwave sterilization was performed to inactivate the spores of biofilms of *Bacillus cereus*. Complete inactivation based on cell membrane destruction by microwaves was achieved within 5 min, and the efficiency of inactivation by microwaves was clearly higher than that by conventional steam autoclaves (Park et al., 2017).

Recently, as a new attempt to sterilize *Bacillus* spores related to foodborne illness and food deterioration using microwave heating or superheated steam has been studied and related commercial facilities have been commercialized and applied in production sites. As such, for commercial development of effective sterilization methods for *Bacillus* spores, research into non-thermal sterilization that combines electrical, physical and optical energy to minimize quality damage by heat is expected to continue.

### Inactivation using combination of physical and chemical treatments

In addition to the above methods, combining various physical preservation methods such as UV irradiation with antibacterial agents such as surfactant components may have a synergistic effect on *Bacillus* spore inactivation (Choi et al., 1997; Elmnasser et al., 2007). The energy required from UV irradiation to inactivate the *B. subtilis* spores was 50% lower in the presence of 1% ethanol extract of *T. japonica* fruit than with UV irradiation alone. Besides, the LD_90_ value, which is the inactivation time to reduce 90% of microorganisms, on the inactivation of the intact *Bacillus* spores shows about 50% decrease in the value, from 0.60 min at UV irradiation alone to 0.32 min at the combined treatment (Cho et al., 2012; Setlow, 2006).

And some studies have shown synergistic inactivation effects of combined PEF with antibacterial chemical extracts such as essential oils, polyphenols against *Bacillus* spores. *B. subtilis* spores was seen with cardamom oil that led to a 3.12-log reduction, and the addition of nisin prior to PEF treatment on *B. cereus* spores in skim milk resulted in a 3-log reduction (Soni et al. 2016).

The combined treatment of physical energy such as heating, high pressure, ultrasound, electric energy, ultraviolet, intense pulsed light and chemical agents such as natural antimicrobial agents with surfactant components can act synergistically and effectively to kill spores. And also, can be a potential non-thermal method on microbial inactivation in various food industry.

As such, research on non-thermal sterilization that combines high intensity electrical, physical and light energy minimizing the quality damage by heating for commercial development of effective sterilization methods for *Bacillus* spores related to foodborne illness and food deterioration is expected to continue.
